# PANCREATIC SOLID-PSEUDOPAPILLARY NEOPLASM IN PATIENTS WITH FAMILIAL ADENOMATOUS POLYPOSIS

**DOI:** 10.1590/0102-672020220002e1718

**Published:** 2023-01-09

**Authors:** José Donizeti MEIRA-JÚNIOR, Gustavo Gonçalves YOGOLARE, Daniel de Paiva MAGALHÃES, Guilherme Naccache NAMUR, Fabio Guilherme CAMPOS, Vanderlei SEGATELLI, Sergio Carlos NAHAS, Jose JUKEMURA

**Affiliations:** 1 Universidade de São Paulo, Faculty of Medicine, University Hospital, Gastroenterology Department, Digestive Surgery Division – São Paulo (SP), Brazil; 2 Universidade de São Paulo, Faculty of Medicine, University Hospital, Pathology Department – São Paulo (SP), Brasil

**Keywords:** beta Catenin, Pancreatectomy, Colorectal Neoplasms, Pancreatic Neoplasms, Adenomatous Polyposis Coli, beta Catenina, Pancreatectomia, Neoplasias Colorretais, Neoplasias Pancreáticas, Polipose Adenomatosa do Colo

## Abstract

**BACKGROUND:**

Solid pseudopapillary neoplasm of the pancreas is an uncommon pancreatic tumor, which is more frequent in young adult women. Familial adenomatous polyposis is a genetic condition associated with colorectal cancer that also increases the risk of developing other tumors as well.

**AIM:**

The aim of this study was to discuss the association of familial adenomatous polyposis with solid pseudopapillary neoplasm of the pancreas, which is very rare.

**METHODS:**

We report two cases of patients with familial adenomatous polyposis who developed solid pseudopapillary neoplasm of the pancreas of the pancreas and were submitted to laparoscopic pancreatic resections with splenic preservation (one male and one female).

**RESULTS:**

ß-catenin and Wnt signaling pathways have been found to play an important role in the tumorigenesis of solid pseudopapillary neoplasm of the pancreas, and their constitutive activation due to adenomatous polyposis coli gene inactivation in familial adenomatous polyposis may explain the relationship between familial adenomatous polyposis and solid pseudopapillary neoplasm of the pancreas.

**CONCLUSION:**

Colonic resection must be prioritized, and a minimally invasive approach is preferred to minimize the risk of developing desmoid tumor. Pancreatic resection usually does not require extensive lymphadenectomy for solid pseudopapillary neoplasm of the pancreas, and splenic preservation is feasible.

## RESUMO

**RACIONAL:**

A neoplasia sólida pseudopapilífera do pâncreas é um tumor pancreático incomum, mais frequente em mulheres jovens. A polipose adenomatosa familiar, por sua vez, é uma condição genética associada a câncer colorretal e que também aumenta o risco de desenvolvimento de outros tumores.

**OBJETIVOS:**

Discutir a associação entre polipose adenomatosa familiar e neoplasia sólida pseudopapilífera, que é bastante rara.

**MÉTODOS:**

Reportamos dois casos de pacientes com polipose adenomatosa familiar, um homem e uma mulher, que desenvolveram neoplasia sólida pseudopapilífera do pâncreas e foram submetidos a ressecção laparoscópica com preservação esplênica.

**RESULTADOS:**

As vias de sinalização da ß-catenina e Wnt tem um papel importante na tumorigênese da neoplasia sólida pseudopapilífera, e sua ativação constitutiva devido a inativação do gene *adenomatous polyposis coli* na polipose adenomatosa familiar pode explicar a relação entre polipose adenomatosa familiar e neoplasia sólida pseudopapilífera.

**CONCLUSÕES:**

A ressecção do cólon deve ser priorizada, com preferência pela abordagem minimamente invasiva para minimizar o risco de desenvolvimento de tumor desmoide. A ressecção pancreática geralmente não requer linfadenectomia extensa para neoplasia sólida pseudopapilífera, portanto, a preservação esplênica é factível.

## INTRODUCTION

Solid pseudopapillary neoplasm of the pancreas (SPN) is an uncommon pancreatic tumor^7^, occurring predominantly in young women aged between 18 and 35 years^14^, with a female-to-male ratio of 7–11:1^2,11^. It has low malignant potential and is usually associated with a favorable prognosis with a long-term disease-free survival of 95%; however, some cases may be locally aggressive and infiltrative, with metastases to the liver, lung, and skin, especially in men^15,18^.

Familial adenomatous polyposis (FAP) is a genetically inherited disease caused by mutations in the adenomatous polyposis coli (APC) or human MUT homologue genes^5^. This syndrome is commonly associated with the development of colorectal cancer and other tumors as well.

To the best of our knowledge, there are only two case reports of FAP in male patients who developed a pancreatic SPN^10,13^ and three cases reported in female patients^9,16,20^ in the literature reviewed.

The aim of this article was to report the third case of FAP and solid-pseudopapillary tumor (SPT) and review the current literature.

## METHODS

We report two cases of patients with FAP who developed SPN of the pancreas and were submitted to laparoscopic pancreatic resections with splenic preservation — one male and one female. The patients signed informed consent, authorizing these records.

## RESULTS

### Case 1

A 54-year-old male was investigated because his daughter was diagnosed with colorectal cancer and colonic polyposis. He was submitted to a colonoscopy, which showed more than 100 polyps throughout the colon. Investigation proceeded with normal upper gastrointestinal endoscopy and computed tomography (CT) of the chest, abdomen, and pelvis.

With this diagnosis, he underwent a laparoscopic total proctocolectomy with ileal J pouch-anal anastomosis and protective ileostomy. Postoperative recovery was uneventful, and the patient was discharged on the 20th postoperative day. Four months later, the ileostomy was closed.

Pathological examination of the surgical specimen demonstrated several adenomatous polyps throughout the colon, two of them demonstrating intramucosal adenocarcinomas. There were 29 lymph nodes dissected; all of them were cancer-free.

**Figure 1:**
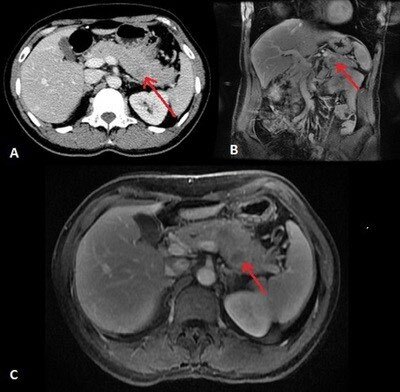
(A) Axial abdominal CT scan showing a hypodense nodule in pancreatic tail; (B) Coronal MRI showing a nodule in pancreatic tail; (C) Axial MRI showing a nodule in pancreatic tail.

One year after the first surgery, computed tomography showed a hypovascular nodule situated in the pancreatic tail. Magnetic resonance imaging with cholangiopancreatography confirmed previous findings. Endoscopic ultrasound-guided fine-needle aspiration was compatible with SPN (Figure 1).

The patient was then submitted to laparoscopic distal pancreatectomy with splenic preservation. Postoperative recovery was uneventful; the surgical drain showed no signs of pancreatic fistulae, and the patient was discharged on the 4th postoperative day after withdrawal of the drain.

**Figure 2:**
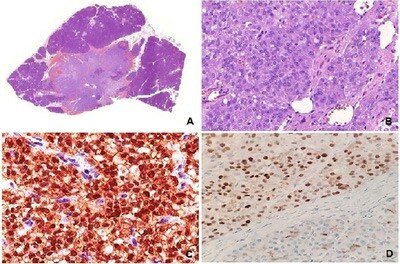
(A) Small, unencapsulated nodular neoplasm (1×, HE); (B) Solid-pseudopapillary microscopic architectural pattern (10×, HE); (C) Nuclear immunoexpression for beta-catenin; and (D) Nuclear immunoexpression for cyclin D1.

In the pathological evaluation, the macroscopic study showed a small (1.2 cm), nonencapsulated neoplasm composed of a firm, brownish tissue with hemorrhagic spots. Microscopically, we observed a neoplasm with a solid and pseudopapillary pattern, composed of cells with vesicular nuclei and abundant vacuolated cytoplasm. Some intracytoplasmic hyaline globules are observed. The immunohistochemical study showed positive immunoexpression for low-molecular-weight cytokeratins, vimentin, progesterone receptor, cyclin D1, CD99 (perinuclear dot pattern) and for nuclear immunoexpression of beta-catenin (Figure 2).

Currently, the patient has regular follow-ups in the outpatient clinic without any complaints or signs of tumor recurrence.

### Case 2

A 34-year-old female presented with diffuse abdominal pain and blood in the stool for 1 year. A colonoscopy demonstrated several polyps throughout her colon, and the diagnosis of FAP was made. Investigation proceeded with an abdominal CT scan, which showed a 4.5 cm heterogenic mass in the pancreatic neck. Upper gastrointestinal endoscopy was normal.

After discussion with the multidisciplinary board, it was decided to initiate the approach with the total proctocolectomy and then proceed with the pancreatic resection.

A laparoscopic total proctocolectomy with ileal J pouch-anal anastomosis and protective ileostomy was performed. Six months later, the ileostomy was closed. A laparoscopic central pancreatectomy with Roux-en-Y pancreaticojejunostomy was performed 10 months later. Postoperative recoveries were uneventful.

Pathological evaluation of the pancreatic specimen showed an SPT of 2.4 cm. The immunohistochemical study showed positive nuclear immunoexpression of beta-catenin.

Currently, the patient has regular follow-ups in the outpatient clinic without any complaints or signs of tumor recurrence.

## DISCUSSION

Classical FAP is caused by inactivation of the APC gene, resulting in constitutive activation of Wnt/ß-catenin signaling, which may be the initial event in the pathogenesis of colorectal cancer^3^. Approximately 25–30% of cases are de novo mutations of the APC gene; therefore, the absence of family history may occur eventually^12^.

FAP is also associated with an increased risk of developing several extraintestinal tumors such as osteomas, desmoid tumor, epidermoid cysts, congenital hypertrophy of the retinal pigment epithelium, fundic gland polyposis, and cancer of the duodenum, thyroid, pancreas, biliary tract, and stomach^6^. Pancreatic tumors are a rare form of extracolonic manifestations of FAP, and most cases of pancreatic tumors in FAP patients are ductal adenocarcinomas. The risk of a FAP patient to develop a pancreatic ductal adenocarcinoma has been estimated to be more than four times compared to that observed in the general population^26^.

SPN’s most common symptoms are pain and sensation of a mass, although up to 15% of patients are asymptomatic^7,18^. Serum markers, such as CA-19.9, alpha-fetoprotein, carcinoembryonic antigen, and CA-125, do not help in the diagnosis of SPN^15,18^. Diagnosis can be confirmed by a CT scan that usually shows a large pancreatic mass with areas of cystic degeneration^4,8^. In small tumors that can mimic well-differentiated neuroendocrine tumor (NET), only the tomographic aspect may not be enough to confirm the diagnosis; in this scenario, endoscopic ultrasonography-guided fine-needle aspiration/biopsy can be useful in identifying the tumor^15,18,19^. In the first presented case, the patient was male and the lesion was small, which made it difficult to confirm the diagnosis with only imaging methods, and the endoscopic ultrasonography-guided fine-needle aspiration was necessary. In the second case, the precise diagnosis was uncertain only with the imaging methods, but the conduct was clearly surgical.

The pathogenesis of SPN is still not fully understood. There is no evidence of estrogen receptors related to tumor pathogenesis, nor any particular role of the p53 gene and k-ras^18,26^. ß-catenin and Wnt signaling pathways have been found to play an important role in tumorigenesis and are consistently positive in about 90% of cases of SPN^1,22^. The somatic activating mutation in exon 3 of CTNNB1 is the only known genetic alteration^1,18,22^. Constitutive activation of Wnt/ß-catenin signaling due to inactivation of APC gene may explain this association between SPT and FAP.

The association between SPN and FAP is very rare. To the best of our knowledge, there are five case reports, three of them reporting female patients^9,16,20^ and two reporting male patients^10,13^. Therefore, we report the third case in the literature of a male patient and the fourth of a female patient with SPT and FAP.

The most common location is the pancreatic tail (35.9%), then the head (34%), and the body (14.8%). Surgery is the main treatment for pancreatic SPT. Adequate treatment offers an excellent prognosis, with a 5-year survival rate over 95%^6,10^. Splenic preservation may be attempted^25^. Extensive lymphatic dissection is not necessary, as SPN is a low-grade malignancy and lymph node involvement is rare^23,25^. For metastatic disease, surgical debulking should be performed, in contrast to other pancreatic malignancies^17,21^. The laparoscopic approach is preferred since it is associated with a lower incidence of desmoid tumor after abdominal surgery^24^.

## CONCLUSIONS

The association between SPN and FAP is very rare. The pathogenesis of this rare tumor is still not clear, but it may be correlated with FAP pathophysiology. Surgical resection offers an excellent cure rate.
